# The SRCIN1/p140Cap adaptor protein negatively regulates the aggressiveness of neuroblastoma

**DOI:** 10.1038/s41418-019-0386-6

**Published:** 2019-07-08

**Authors:** Silvia Grasso, Davide Cangelosi, Jennifer Chapelle, Melissa Alzona, Giorgia Centonze, Alessia Lamolinara, Vincenzo Salemme, Costanza Angelini, Alessandro Morellato, Andrea Saglietto, Federico Tommaso Bianchi, Sara Cabodi, Iris Chiara Salaroglio, Federica Fusella, Marzia Ognibene, Manuela Iezzi, Annalisa Pezzolo, Valeria Poli, Ferdinando Di Cunto, Alessandra Eva, Chiara Riganti, Luigi Varesio, Emilia Turco, Paola Defilippi

**Affiliations:** 1 Department of Molecular Biotechnology and Health Sciences, University of Torino, 10126 Torino, Italy; 2 Laboratory of Molecular Biology, Giannina Gaslini Institute, 16147 Genova, Italy; 3 Department of Medicine and Aging Science, Center of Excellence on Aging and Translational Medicine (CeSi-Met), G. D’Annunzio University, Chieti-Pescara, Italy; 4 Cardiology Division, Department of Medical Sciences, University of Torino, 10126 Torino, Italy; 5 Neuroscience Institute Cavalieri Ottolenghi, Regione Gonzole 10, 10043 Orbassano (TO), Italy; 6 Department of Oncology, University of Torino, 10126 Torino, Italy; 7 Laboratorio Cellule Staminali Post Natali e Terapie Cellulari, Istituto Giannina Gaslini, Genova, Italy

**Keywords:** Paediatric cancer, Molecular biology

## Abstract

Neuroblastoma is the most common extra-cranial pediatric solid tumor, responsible for 13–15% of pediatric cancer death. Its intrinsic heterogeneity makes it difficult to target for successful therapy. The adaptor protein p140Cap/SRCIN1 negatively regulates tumor cell features and limits breast cancer progression. This study wish to assess if p140Cap is a key biological determinant of neuroblastoma outcome. RNAseq profiles of a large cohort of neuroblastoma patients show that SRCIN1 mRNA levels are an independent risk factor inversely correlated to disease aggressiveness. In high-risk patients, CGH+SNP microarray analysis of primary neuroblastoma identifies SRCIN1 as frequently altered by hemizygous deletion, copy-neutral loss of heterozygosity, or disruption. Functional experiments show that p140Cap negatively regulates Src and STAT3 signaling, affects anchorage-independent growth and migration, in vivo tumor growth and spontaneous lung metastasis formation. p140Cap also increases sensitivity of neuroblastoma cells to doxorubicin and etoposide treatment, as well as to a combined treatment with chemotherapy drugs and Src inhibitors. Our functional findings point to a causal role of p140Cap in curbing the aggressiveness of neuroblastoma, due to its ability to impinge on specific molecular pathways, and to sensitize cells to therapeutic treatment. This study provides the first evidence that the *SRCIN1*/p140Cap adaptor protein is a key player in neuroblastoma as a new independent prognostic marker for patient outcome and treatment. Altogether, these data highlight the potential clinical impact of *SRCIN1*/p140Cap expression in neuroblastoma tumors, in terms of reducing cytotoxic effects of chemotherapy, one of the main issues for pediatric tumor treatment.

## Background

Neuroblastoma (NB) is a complex disease with different outcome, responsible for a large proportion of cancer death in childhood [1, 2]. Primary NB originate from genetic abnormalities in neural crest-derived sympathoadrenal cells [1]. The International Neuroblastoma Staging System (INSS) defines 5 stages of NB progression [3], from Stages 1 and 2 (localized tumor, without lymph nodes involvement) to Stage 3 and Stage 4 (metastatic disease, with dissemination in distant organs). Stage 4S specifies a metastatic disease in children under the age of 1 year, which may undergo spontaneous regression usually associated with 90% survival rate at 5 years. The therapeutic intervention in NB (surgery for stages 1 and 2, and multiple-agent chemotherapy [4, 5]), does not increase substantially the favorable outcomes [4], indicating that additional therapeutic strategies with increased effectiveness and reduced toxicity are an urgent need.

Established markers of poor prognosis for NB include segmental chromosome abnormalities (chromosomes 1p, 3p, 4p, 11q loss and 1q, 2p, 17q gains), and DNA ploidy (International Neuroblastoma Risk Group (INRG): http://inrgdb.org/) [6, 7]. At molecular levels, *ALK* is the most frequently mutated gene in hereditary familial NB, in 6–11% of NB cases [6–8]. *MYCN* proto-oncogene amplification occurs in 20% of NB, in poor-prognosis patients resistant to therapy [9–12]. In addition, driver mutations in *LIN28B* (Lin 28 homolog B) [13], or *PHOX2B* (Paired-like Homeobox 2b) [14], have been reported.

The p140Cap adaptor protein [15], also known as SNIP [16], plays a causal role in HER2-related breast cancer progression, and its expression is associated with good prognosis [17]. Of note, p140Cap impairs tumor growth and metastasis formation, interfering with Src kinase [18] and Rac GTPases [17] activation.

p140Cap in differentiated neurons controls synaptic plasticity [19, 20], and regulates GABAergic synaptogenesis and development of hippocampal inhibitory circuits [21]. Taking into account the functional role of p140Cap in differentiated neural cells, we set out to tackle its relevance in human NB, analyzing the expression of the p140Cap encoding gene *SRCIN1*, its genomic profile and its causal role in NB aggressiveness. We show that *SRCIN1* mRNA is an independent prognostic risk factor for NB, and that the *SRCIN1* gene is frequently altered in high stage patients. The p140Cap protein plays a key role in curbing the aggressiveness of the NB tumors, counteracting oncogenic signaling pathways and resulting in impaired tumor progression and enhanced sensitivity to treatments.

## Materials and methods

### Gene expression dataset

We used a dataset containing the gene expression profile and related clinical information of 498 primary tumors of NB patients measured by the Illumina HiSeq 2000 RNAseq platform [22]. The dataset is available to registered users in the R2: Genomic Analysis and Visualization Platform (http://r2.amc.nl). The risk factors considered were: (1) tumor stage (st), defined as st1, st2, st3, st4, or st4s according to the International Neuroblastoma Staging System (INSS) [2, 3] *MYCN* oncogene amplification, (3) age at diagnosis before and after 12 months. Good and poor outcome was defined as the patient’s alive or dead status 5 years after diagnosis.

### NB cell lines

ACN (HTL-96020), LAN-1 and IMR-5 cells were obtained by ICLC-Interlab Cell Line Collection at IRCCS AOU San Martino-IST, Genova, Italy. Sk-N-Be(2), SH-SY-5Y, SK-N-SH, IMR-32, and HTLA-230 cells were obtained from ATCC (LGC Standards S.r.l., Italy Office, Italy). LAN-1 cells came from DSMZ (Braunschweig, Germany). ACN cells were cultured in DMEM supplemented with 10% Fetal Calf serum (FCS). Sk-N-Be(2), SK-N-SH and SH-SY-5Y cells were cultured in 1:1 mix of MEM:F12 Nutrient mix, supplemented with 10% FCS, 1% NMNEEA, 1% Sodium Pyruvate and 2 mM Glutamine. IMR-5, IMR-32, LAN-1 and HTLA-230 cells were cultured in RPMI supplemented with 10% FCS. Culture media were from Invitrogen (Carlsbad, CA, USA). FCS was from EuroClone (Pero, Milano, Italy). The genomic identity of each line was regularly confirmed using array-CGH, and cell lines were routinely tested to confirm the lack of mycoplasma contamination.

### Patients and tumor samples

We recovered a retrospective series of 225 primary NB of all stages, with 17q gain according to the INSS, diagnosed in the period from January 1995 to December 2017 in Italy at one out of 23 centers of the Italian Association of Pediatric Hematology and Oncology (AIEOP). Frozen tumor samples from these patients were analyzed by a-CGH and SNP-array. The data are stored in the BIT-Gaslini Biobank of Istituto Giannina Gaslini, Genova, Italy. Tumor samples were obtained before treatment at the time of diagnosis. Tumor DNAs were extracted from fresh NB tissue using the QIAamp DNA Extraction Kit (Qiagen, Hilden, Germany), according to the manufacturer’s instructions. Tumor content was confirmed by review of hematoxylin and eosin stained tumor sections by the local pathologists.

The patients data derived from Italian Neuroblastoma Registry (INBR) of AIEOP. The clinical characteristics of NB patients are collected in pseudo-anonymized manner and stored in a secure system at the Epidemiology and Biostatistics Unit of the Istituto Giannina Gaslini.

### Genomic profile analysis

DNA from NB cell lines and primary tumors was tested by high-resolution oligonucleotide a-CGH and SNP-array using the 4 × 180K Kit (Agilent Technologies) with a mean resolution of approximately 25 kb. SNP-array and oligo-array data were analyzed with Genomic Workbench 7.0.40 software (Agilent). To make aberration calls, an aberration detection algorithm ADM-1 was used at threshold 10, and an aberration filter was set at 2 for the minimum number of probe region, and at 1 for minimum absolute average log2 ratio to reduce false positives. Chromosome positions were determined using GRCh/hg19 (UCSC Genome Browser, http://genome.ucsc.edu, Feb. 2009 release). The quality of the test was assessed on the strength of the QCmetrics values. Polymorphisms (http://projects.trag.ca/variation/) were not included because considered normal variants.

### Retrovirus production and cell infection

The p140Cap cDNA was cloned into pBabe-puro vector, as described in [17]. The retrovirus particles were produced by the calcium phosphate transfection of Platinum Retroviral Packaging Cell Lines (Cell BioLabs), in 10 cm dishes. Briefly, 48 h (h) after transfection, supernatant was collected, filtered through a 45 μm syringe filter and added directly to sub-confluent cells. After 48 h, cells were washed and cultured with a selection medium containing puromycin (Sigma) at a final concentration of 1 µg ml^−1^. The efficiency of infection was controlled by western blot (WB) analysis (see below). Individual clones were isolated 20 days after the start of the selection. Four individual positive clones were pooled together to rule out clonal artifacts.

Cells expressing p140Cap were infected with the retrovirus construct LZRSpBMN-Z-GFP expressing constitutive active STAT3 (STAT3C) [23] and processed as above.

### Immunoblotting and antibodies

Proteins were extracted using RIPA buffer (150 mM NaCl, 1% NP-40, 0.5% Sodium Deoxycholate, 0.1% SDS and 50 mM Tris pH. 7,5) with 1 mM PMSF, 10 mM NaF, 2 mM NaVO_3_ and Protease Inhibitor cocktail (cat.no. 04-693-1160-001, Roche, USA). For analysis of phosphorylated histones, extracts were prepared in boiling 2% SDS buffer (in 150 mM Tris pH 6,8). Western Blot (WB) analysis was performed as previously described in [17]. The following antibodies were used: mouse monoclonal antibodies (Mab) to p140Cap already characterized in [17] (1:500), anti phospho-p130Cas (Tyr410; #4011, 1:1000), anti phospho-Src (Tyr416; #2101, 1:1000), anti phospho-JAK2 (Tyr1007/1008; #3776S, 1:1000), anti γH2AX (Ser139; #2577, 1:1000) and anti H2AX (#2595, 1:1000) from Cell Signaling, Beverly, MA; anti GAPDH (MAB374, 1:8000) from Millipore, Billerica, MA, USA; anti p130Cas (cat.no 610272, 1:2500) from BD Transduction Laboratories, Franklin Lakes, NY; anti Src (B-12, 1:1000) and anti JAK2 (sc-278, 1:1000) from Santa Cruz Biotechnologies, Palo Alto, CA, USA; anti Tubulin (T5168, 1:8000) from Sigma-Aldrich Co, Italy. Secondary antibodies conjugated with peroxidase and nitrocellulose membranes were purchased from GE Healthcare (Buckinghamshire, UK). When appropriate, the membranes were stripped according to manufacturers’ recommendations and re-probed.

### Proliferation and anoikis assays

Mock and p140 cells (1 × 10^5^) were seeded in 6-well plates. At 24, 48, 72 and 96 h, cells were trypsinized and quantified using a Burker chamber (5 squares for each sample were counted, giving the average of cell number). For anoikis assays, cells were detached and kept in suspension in serum-free media. For apoptosis detection, cells were stained with APC Annexin V probe (1:200) (Biolegend, San Diego, CA, USA) for 15 min at room temperature, followed by Propidium Iodide staining (P4864, Sigma-Aldrich Co, Italy). Flow cytometric analyses were carried out on a FACSCalibur using CellQuest Software (Becton Dickinson). WB for Bcl2 expression was performed with anti Bcl2 antibodies (N-19 sc-492, 1:1000) from Santa Cruz Biotechnologies, Palo Alto, CA, USA). At least three independent experiments, with three replicates for each condition, were performed.

### Soft agar assay

Anchorage-independent growth was performed in soft agar as described in [18]. Briefly, 5 × 10^4^ cells were suspended in complete DMEM containing 1.3% Agarose Low-gelling temperature (Sigma Life Science, UK, A9141-100G) and seeded into 6 well plates containing 2 ml layer of solidified 2% Agarose (Sigma Life Science, UK, A9539-500G). After 21 days, colonies were detected using a 4% of Crystal Violet solution containing 1% of Ethanol. Images of colonies were acquired with a ×1.8 and ×4 objectives using a Leica MZ125 stereo microscope fitted with a Nikon Coolpix colour digital microscope camera. At least three independent experiments, with three replicates for each condition were performed.

### Wound healing assay

Mock and p140 cells were plated in 6-well plates and allowed to grow at confluence. Upon vertical and horizontal linear scratches, using a 200 µl pipette tip, cells were washed twice with PBS and maintained in serum-free medium. Images were acquired at 0, 24, and 48 h post scratch with ×10 objective Carl Zeiss microscope. Lines were drawn to pinpoint scratch boundaries. Horizontal width of the scratch was measured using Axio-Vision coordinates. For quantification, three replicate wells for each condition were analyzed in at least 3 independent experiments.

### Transient silencing of p140Cap in SH-SY-5Y cells

Transient transfections of ON-TARGET plus human SRCIN1 small-interfering RNA (siRNA) or ON-TARGET plus non-targeting siRNA (Dharmacon RNAi, GE Healthcare, Buckinghamshire, UK) were performed with Lipofectamine 2000 (Invitrogen, USA) according to manufacturer’s protocol, as already described in [17]. This patented approach is the best strategy to prevent off-target effects caused by both the sense and antisense strands while maintaining high silencing potency. Briefly, cells were plated on six-well plates and transfected at 80% confluency. Either 5 µl of 20 µM p140Cap siRNA or non-targeting siRNA were added to each well, and cells were incubated for 48 h at 37 °C in a humidified CO2 incubator.

### Heterotopic xenograft tumour growth

Six-week-old male NSG, NOD/SCID/gamma mice (NOD.Cg-Prkdcscid Il2rgtm1Wjl/SzJ) were obtained from Charles River Laboratories (Calco, Italy; *n* = 5 per group for each experiment) and treated in accordance with the European Community guidelines. Briefly, 2 × 10^5^ cells (in 0.2 ml of PBS) were injected subcutaneously into the dorsal region of male NSG mice. The size of the tumors was evaluated twice a week using digital calipers in blind experiments. Mice were sacrificed when tumor reached a 10 mm diameter.

### Histology, immunohistochemistry and immunofluorescence of tissue samples

Tissue samples were fixed in 10% neutral buffered formalin and embedded into paraffin or fixed in 4% PFA and frozen in a cryo-embedding medium (OCT, BioOptica); slides were cut and stained with Hematoxylin (BioOptica) and Eosin (BioOptica) for histological examination. For immunohistochemistry, slides were deparaffinized, serially rehydrated and, after the appropriate antigen retrieval procedure, stained with mouse monoclonal anti-human Ki67 (M7240, Dako) or mouse monoclonal anti-p140Cap antibody (see above), followed by the appropriate secondary antibodies. Immunoreactive antigens were detected using streptavidin peroxidase (Thermoscientific) and the DAB Chromogen System (Dako). After chromogen incubation, slides were counterstained in Hematoxylin (BioOptica) and images were acquired by Leica DMRD optical microscope (Leica). The percentage of Ki67 positive cells was evaluated on digital images of 5 tumors per group (5–8 × 200 microscopic fields per tumor); clear brown nuclei were regarded as positive cells and the percentage of positive cells (number of positive cells/total cells ×100) was calculated for each field, by two pathologists, independently. For adrenal gland staining, mouse monoclonal anti-p140Cap antibody and mouse monoclonal anti-human Chromogranin A (M 0869, Dako) were used.

For immunofluorescence, cryostat sections were air-dried, fixed in ice-cold acetone for 10 min and incubated with the following primary antibodies: rat monoclonal anti-CD31 (550274, BD Pharmingen) mixed with rat monoclonal anti-CD105 (550546, BD Pharmingen, San Diego, CA), mouse monoclonal anti-α Smooth Muscle Actin (Clone 1A4 1:1000) from Sigma, Saint Louis, Missouri) and rabbit polyclonal anti-NG2 (ab5320, Millipore) followed by secondary antibodies conjugated with Alexa 546 and Alexa 488 (Invitrogen, Life Technologies, Monza, Italy), respectively. Nuclei were stained with DRAQ5 (Alexis, Life Technologies, Monza, Italy). Image acquisition was performed using Zeiss LSM 510 META confocal microscope. The extent of pericyte coverage on vessels was evaluated on digital images of 3–4 tumors per group (3 × 200 microscopic fields per tumor), with Adobe Photoshop by selecting endothelia (red pixel) and pericytes (green pixel) with the Magic Wand Tool and reporting the number of pixels indicated in the histogram window (green pixel/red pixel ×100).

### Spontaneous lung metastasis evaluation

For spontaneous lung metastasis assay, mice were sacrificed when tumor reached a 10 mm diameter. Lungs were explanted, fixed in 10% neutral buffered formalin and paraffin-embedded. To optimize the detection of microscopic metastases and ensure systematic uniform and random sampling, lungs were cut transversally, to the trachea, into 2 mm thick parallel slabs with a random position of the first cut in the first 2 mm of the lung, resulting in 5–8 slabs for lung. The slabs were then embedded cut surface down, sections were stained with Hematoxylin and Eosin (BioOptica, Milan, Italy) and the number of metastases was evaluated by two pathologists, independently.

### Cell viability assay

Mock and p140 cells were plated in 24-well plates and allowed to grow until 70–80% confluence, then starved overnight in a serum-free medium. Chemotherapeutic drugs (carboplatin, ciclophospamide, doxorubicin, etoposide, and vincristine) were all obtained from Sigma. The compounds were dissolved in dimethyl sulfoxide (DMSO) and added to cell medium at the final concentration indicated in the text. After 24, 48, and 72 h of treatment, cells were washed with PBS and stained with 5% w/v crystal violet solution in 66% v/v methanol, then washed once in deionized water. Absorbance was read at 570 nm, using a Packard EL340 microplate reader (Bio-Tek Instruments, Winooski, VT). The absorbance of untreated cells was considered as 100% viability; the results were expressed as percentage of viable cells vs. untreated cells. p140-silenced SH-SY-5Y cells were treated with doxorubicin or etoposide 24 h after transfection. Cell viability was tested after 48 h.

### Immunofluorescence, microscopy and cell image analysis

For p140Cap immunostaining, cells were fixed at RT with 2% paraformaldehyde (PFA) for 10 min. Cells were then permeabilized in 0.25% Triton-X-100 in PBS for 10 min, saturated with 1% BSA and 0.25% Triton-X-100 in PBS for 30 min and incubated with primary p140Cap antibody (1:500, see above) at RT for 90 min. Primary antibody was detected with anti-mouse Alexa Fluor 488 (Molecular Probes, Invitrogen, Life Technologies, Monza, Italy), used at 1:500 dilution for 1 h. For actin staining, Fluorescein Isothiocyanate Labeled Phalloidin was used (P5282; 1:500) from Sigma. Counterstaining was performed with the DNA dye DAPI (Sigma) at 0.5 μg/ml DAPI for 5 min and washed with PBS. For gamma H2AX immunostaining, cells were treated 10 min at RT using CSK buffer (300 mM Sucrose, 100 mM NaCl, 3 mM MgCl2 in double distilled H2O), 0.7% Triton-X-100, and fixed 10 min at RT using PFA 4%. Cells were then permeabilized in 0.1% Triton-X-100 in PBS for 5 min, saturated in 5% BSA in PBS for 30 min and incubated with the primary gamma H2AX (1:300, see above) antibody for 1 h at RT. The primary antibody was detected with an anti–rabbit Alexa Fluor 488 (Molecular Probes, Invitrogen, Life Technologies, Monza, Italy), used at 1:500 dilution for 30 min. Finally, cells were counterstained with 0.5 μg/ml DAPI for 5 min and washed with PBS. Images were acquired using an Eclipse 80i-ViCO system (Nikon, Japan). Fixed cells were imaged using a Plan Fluor 40×/1.3 NA oil immersion objective. All the images were analyzed by using Fiji Software (an image processing package distribution of ImageJ, USA). Foci were quantified using “Find Maxima” after the appropriate setting of the threshold and noise.

### Statistical analysis

Overall-Survival (OS) and Event-Free-Survival (EFS) curves were plotted by the Kaplan-Meier method and compared by the log-rank test. *P*-values were corrected for multiple hypotheses testing by Bonferroni method. Mean comparison was assessed by unpaired Student *t* test. Multivariate analysis by Cox proportional regression models tested the prognostic value of *SRCIN1* in the context of concomitant effects of other common prognostic factors for NB (Age at diagnosis, INSS stage and MYCN status).

Results of three independent experiments are reported as mean ± standard error of the mean (SEM). Statistically significant differences were evaluated using unpaired t-tests and Welch’s unequal variances t test for the number of foci per cells. Error bar: SEM. Results were considered significant when *P*-value < 0.05. Statistical tests used the Survival R package (R 3.1.2) or GraphPad Prism version 6.0 for Windows (GraphPad Software, San Diego California USA, www.graphpad.com). The CalcuSyn software (www.biosoft.com/w/calcusyn.htm) was used to calculate the CI (combination index to reduce viable cells to 50%) [24] and the DRI50 (the dose-reduction necessary to decrease viable cells to 50%).

## Results

### Expression and genomic profiling of *SRCIN1* in human NB

**Fig. 1:**
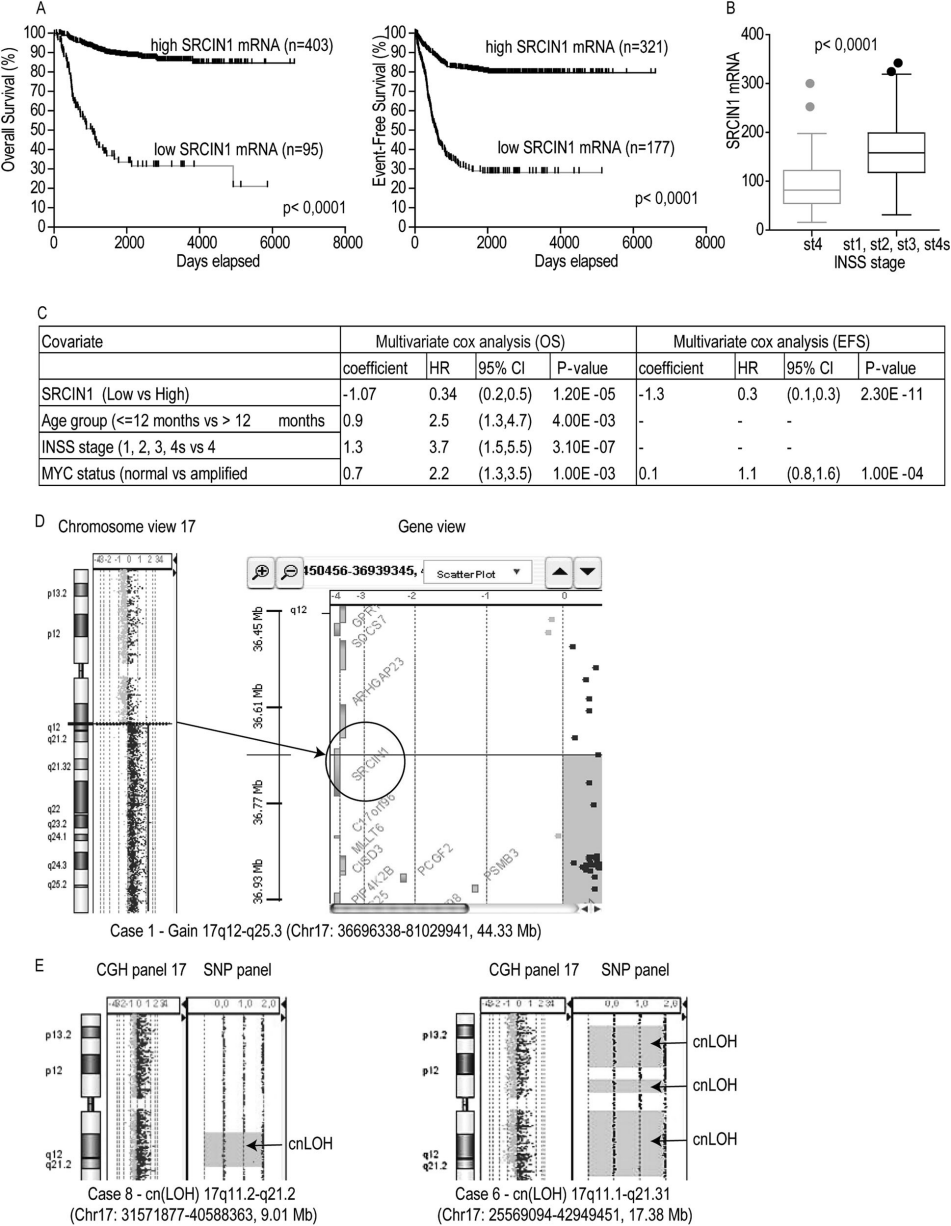
Stratification by *SRCIN1* mRNA expression in primary NB patients and *SRCIN1* gene status. **a** Kaplan-Meier curves for overall (left panel) and event-free (right panel) survival stratified by *SRCIN1* expression in a cohort of 498 NB patients. Cut off for high or low SRCIN1 expression was chosen by Kaplan-Meier scan method. Survival curves were compared by log-rank test. P-values were corrected for multiple hypotheses testing by Bonferroni method. Each plot reports the corrected *P*-value (*P*). Corrected *P*-values lower than 0.05 were considered statistically significant. The number of patients with high or low expression of *SRCIN1* mRNA is reported in every curve. **b** Box and whisker plot for the expression of *SRCIN1* mRNA in the two risk groups defined by INSS stages (st1, st2, st3, st4s vs. st4). The significance of the mean expression was measured by unpaired Student *t*-test. A *P*-value lower than 0.05 was considered significant. **c** Multivariate Cox regression analysis for overall survival (OS) and event-free survival (EFS). The prognostic value of *SRCIN1* mRNA expression (high and low) was tested in the context of known risk factors: age at diagnosis (>12 months vs <12 months) MYCN amplification (normal vs. amplified) INSS stages (st1, st2, st3, st4s vs. st4). Cut off for high or low SRCIN1 expression was chosen by Kaplan-Meier scan method. Cox regression coefficient (coefficient), hazard ratio (HR), 95% of confidence interval (95% CI) and *P*-value are shown for each variable in the OS and EFS panel. Significant *P*-values are lower than 0.05 (OS: HR 0.34 95% CI 0.2–0.5 *P* < 0.0001; EFS: HR 0.27 95% CI 0.1–0.4, *P* < 0.0001). **d** Copy number analysis in NB patient Case 1. Chromosome View: array-CGH identified a 17q12-q25.3 duplication of 44.33 Mb. Gene View: the circle shows that the *SRCIN1* gene is truncated at the breakpoint in 17q12. Setting for CGH aberration calling: ADM-1, threshold 6, minimum of 8 probes, ≥0.1 log ratio. **e** Copy number and copy neutral LOH analysis in Case 8 and Case 6. CGH panels: histogram of the CGH probes log ratio distributions showing the normal copy region of 17q. SNP panels: the grey dots represent the SNP distribution. Copy-neutral LOH (Loss of Heterozygosity) region containing *SRCIN1* gene was detected in chromosome 17 for the case 8 and 6. For case 6 three cn-LOH regions were detected. An LOH call appears as a large grey shaded area. Note the lack of probes that correspond to a copy number of heterozygous signals

The impact of *SRCIN1* expression on NB patient outcome was studied on the R2 platform, containing expression profiles and clinical information of 498 NB cases. We analyzed the relationship between *SRCIN1* mRNA expression and disease evolution by Kaplan-Meier analysis, with respect to overall survival (OS) and event free survival (EFS). Cut off of 68.2 and 101.5 selected by Kaplan-Meier scan method for OS and EFS, respectively, separated patients into two subgroups with high or low *SRCIN1* expression. High expression of *SRCIN1* was associated with good prognosis (403 patients) whereas low expression was observed in 95 poor prognosis patients (log rank test with *P* < 0.0001) (Fig. 1a, left panel). Moreover, high *SRCIN1* expression was found in event-free (321 patients) while a low expression was significantly associated with reduced event-free survival (177 patients) (*P* < 0.0001, Fig. 1a, right panel). We concluded that *SRCIN1* is a prognostic risk factor for NB.

Alternatively, the cohort was split into two groups based on the INSS classification: stages st1, st2, st3, st4s, with no or low metastatic diffusion; st4, in whom the cancer has spread to distant sites. St4 tumors had a significantly lower expression of *SRCIN1* mRNA relative to the st1, st2, st3, st4s group (*P* < 0.0001, Fig. 1b), supporting the conclusion that *SRCIN1* expression is higher in patients with low metastatic spread.

The multivariate Cox analysis including other known risk factors (*MYCN* amplification, INSS stage, and age at diagnosis) showed that *SRCIN1* expression was an independent favorable prognostic factor, both in terms of OS and EFS, with low Hazard Ratio (HR) and highly significant *P*-value (Fig. 1c). As expected, INSS stage was also a strong prognostic indicator. Overall, *SRCIN1* mRNA expression correlates with good outcome and is an independent prognostic marker for NB.

The *SRCIN1* gene is located on chromosome 17q12, a region frequently altered in NB, and associated with poor prognosis. Indeed, the 17q gain, the most frequent chromosome imbalance occurring in 50–70% of all high stage NB, associates with poor prognosis as an independent indicator of adverse outcome [25–28]. The status of the *SRCIN1* gene in NB was assessed by high-resolution oligonucleotide a-CGH and SNP-array on 225 NB primary tumors of all stages with 17q gain. In four NB tumors *SRCIN1* was hemizygously deleted, in three was subjected to copy-neutral Loss Of Heterozigosity (cn-LOH) and in ten was disrupted because located at breakpoint of 17q segment involved in generation of 17q gain (Table 1). Figures 1d, e show representative plots of the chromosome status. However, the OS differences between patients harbouring these alterations did not reach statistical significance (*P* = 0.5), likely due to the small size of the cohort.

**Table 1:** *SRCIN1* loss/cn-LOH or disruption in the breakpoint on 17 NB patients

NB patients	*SRCIN1* gene status	Chromosomal coordinates
Case 1	disrupted in the breakpoint	Chr17: 36696338-81029941 Cytoband: 17q12-q25.3 Size: 44.33 Mb
Case 2	disrupted in the breakpoint	Chr17: 36694901-80943345 Cytoband: 17q12-q25.3 Size: 44.24 Mb
Case 3	loss	Chr17: 25311574-36777884 Cytoband: 17q11.1-q12 Size: 11.46 Mb
Case 4	disrupted in the breakpoint	Chr17: 36696338-80969424 Cytoband: 17q12-q25.3 Size: 44.27 Mb
Case 5	disrupted in the breakpoint	Chr17: 36696279-81029941 Cytoband: 17q12-q25.3 Size: 44.33 Mb
Case 6	copy neutral LOH	Chr17: 25569094-42949451 Cytoband: 17q11.1-q21.31 Size: 17.38 Mb
Case 7	copy neutral LOH	Chr17: 29149425-45297941 Cytoband: 17q11.1-q21.31 Size: 16.14 Mb
Case 8	copy neutral LOH	Chr17: 31571877-40588363 Cytoband: 17q11.2-q21.2 Size: 9.01 Mb
Case 9	loss	Chr17: 25278114-37876263 Cytoband: 17q11.1-q12 Size: 12.59 Mb
Case 10	loss	Chr17: 25278114-68301170 Cytoband: 17q11.1-q24.3 Size: 43.02 Mb
Case 11	disrupted in the breakpoint	Chr17: 36696279-81029941 Cytoband: 17q12-q25.3 Size: 44.33 Mb
Case 12	disrupted in the breakpoint	Chr17: 36696338-81029941 Cytoband: 17q12-q25.3 Size: 44.33 Mb
Case 13	disrupted in the breakpoint	Chr17: 36740844-80943189 Cytoband: 17q12-q25.3 Size: 44.20 Mb
Case 14	disrupted in the breakpoint	Chr17: 36740903-80993001 Cytoband: 17q12-q25.3 Size: 44.25 Mb
Case 15	loss	Chr17: 25278114-81029941 Cytoband: 17q11.1-q25.3 Size: 55.75 Mb
Case 16	disrupted in the breakpoint	Chr17: 36672992-77470237 Cytoband: 17q12-q25.3 Size: 40.79 Mb
Case 17	disrupted in the breakpoint	Chr17: 36694044-81099040 Cytoband: 17q12-q25.3 Size: 44.40 Mb

### p140Cap negatively affects tumorigenic features

**Fig. 2:**
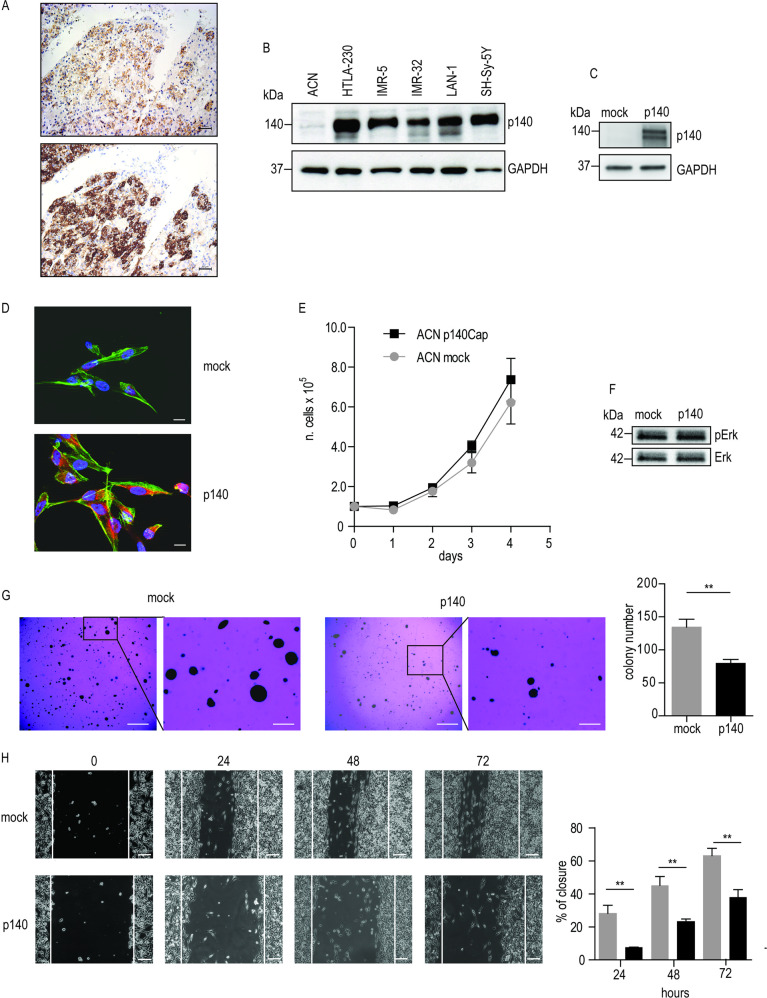
p140Cap expression affects anchorage-independent growth and cell migration. **a** Immunohistochemical analysis of p140Cap expression in human neonatal adrenal gland. p140 staining is visible in the chromaffin cells of the adrenal medulla (upper panel), as confirmed by the Chromogranin A staining (lower panel). Scale bar: 50 µM. **b** p140Cap expression in NB cell lines. Equal amounts of proteins from the indicated cell lines were processed as in (**a**). **c** p140Cap expression in ACN cells upon viral infection. WB analysis was performed on a pool of clones expressing p140Cap. **d** Immunofluorescence analysis of p140Cap in mock versus p140 ACN cells. Cells were plated on glass coverslips and immunofluorescence was performed with FITC-phalloidin (green) and p140Cap antibodies, followed by the appropriate secondary antibodies (red). Scale bar: 10 µM. **e** Cell proliferation of mock and p140 ACN cells. Cells were seeded into 6-well plate as 100.000 cells/well. Cells were detached and manually counted in Burker chambers on triplicate wells every day. Three independent experiments were performed **f**. pErk1/2 MAPK activation by WB analysis. Extracts of mock and p140 cells in standard culture condition were analyzed with antibodies to pErk1/2 and ERk1/2 for loading control. **g** Representative images of Soft Agar assay. Upon 21 days of culture, the number of mock and p140 colonies was quantified by ImageJ software after staining with Crystal Violet. Results were shown as mean ± SEM of three independent experiment (right panel). Unpaired *t*-test **P < 0.01. Scale bar: 0.5 mm (left), with enlargement to 100 µM (right). **h** Wound Healing assay. Mock and p140 cells were seeded into 24-well plates. At confluence scratch was performed (time 0) and pictures were taken every 24 h. Red lines show the width of the scratch at time 0. The percentage of Wound Healing Closure are presented as mean ± SEM of three separate experiments (unpaired *t*-test *P < 0.05). Scale bar 100 µm

The above data provided us with the testable hypothesis that the expression of the *SRCIN1* gene product, the p140Cap adaptor protein, may attenuate the intrinsic biological aggressiveness of NB tumors. NB tumors originate from the developing sympathetic nervous system, and are located in sympathetic ganglia and adrenal glands. We detected p140Cap by immunostaining in the medulla of normal human neonatal adrenal glands (Fig. 2a), indicating that p140Cap is present in the main site of origin of NB tumors. Moreover, in a panel of human NB cell lines, *SRCIN1* gene was lost in ACN, single copy in SH-SY-5Y, IMR-32, and HTLA-230, gain in LAN-1, and subjected to cn-LOH in SK-N-SH cells (Supplementary Fig. S1). Indeed, p140Cap is expressed in all NB cell lines but not in ACN, a neuroblast-like cell line derived from bone marrow metastasis [29] (Fig. 2b), and undetectable in SK-N-SH cells (Supplementary Fig. S1b).

The lack of p140Cap expression in ACN cells renders them suitable to address whether p140Cap gain of function in a NB context may affect tumorigenic phenotype. Pool of mock and p140 cells, obtained by retroviral infection and single cell cloning (Fig. 2c, d), proliferated similarly under standard culture condition (Fig. 2e), with similar Erk1/2 phosphorylation levels (Fig. 2f). However, p140 cells displayed a significantly reduced number of colonies upon 21 days of culture in soft-agar assays (Fig. 2g). In a Wound Healing assay, p140 cells were significantly impaired in migration, leaving the scratch still open at 72 h (lower panels), while mock cells closed the scratch in 48–72 h (Fig. 2h). Overall, p140Cap may limit anchorage-independent growth and migration of NB cells, consistent with the causal involvement of p140Cap in counteracting NB aggressive tumor properties.

### p140Cap impairs the Src/p130Cas and the STAT3/Jak2 signaling pathways

**Fig. 3:**
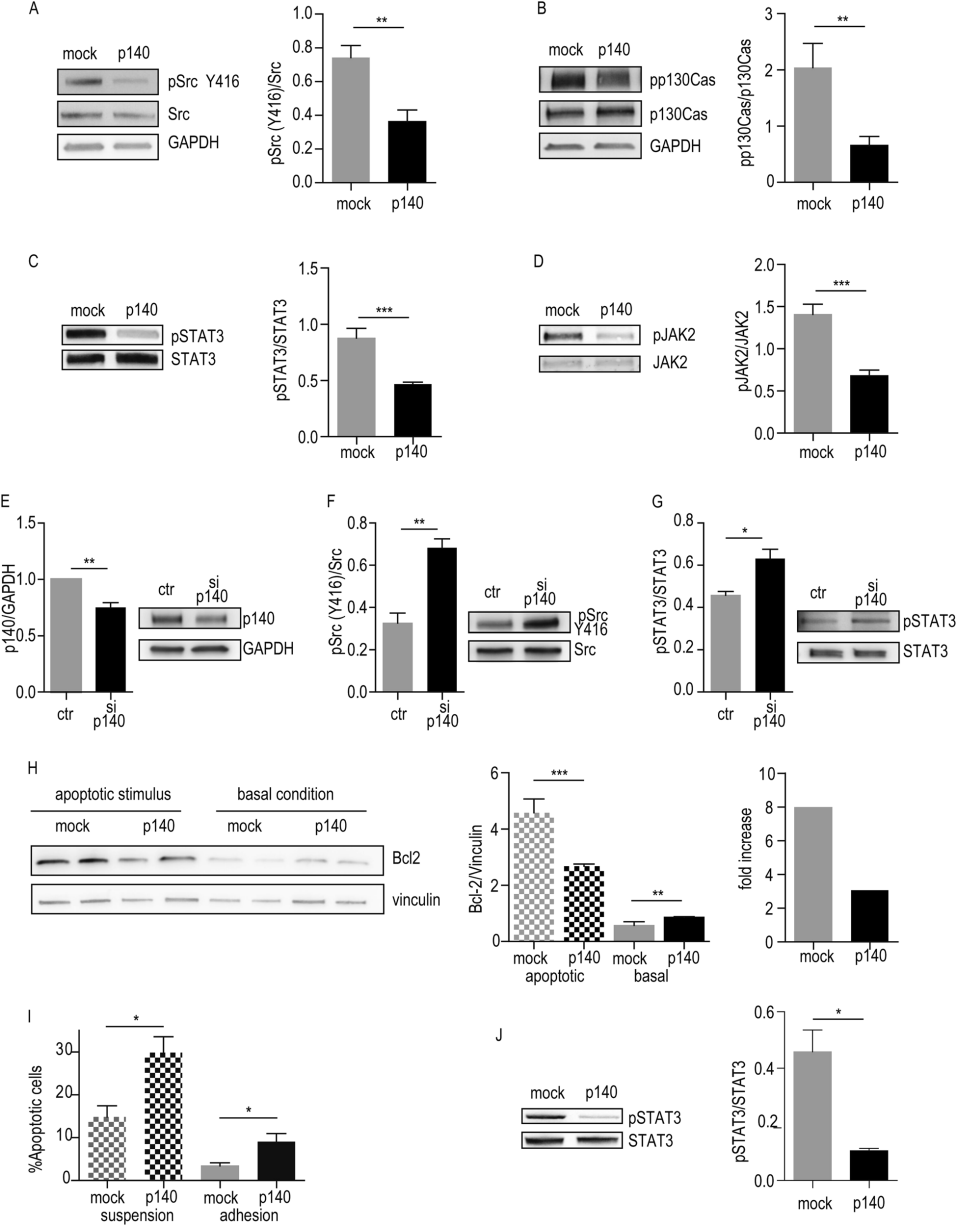
p140Cap affects signaling pathways in NB cells. Cell extracts were run on 4–15% pre-casted SDS-PAGE and analyzed as follows. **a** Mock and p140 ACN cells. Src activation was evaluated by WB analysis of Tyr 416 phosphorylation (Y416) and Src protein level as loading control. Antibodies to GAPDH (as loading control) were used. Quantification on the right is the ratio between phosphorylated Src and total Src protein from three independent experiment, as mean ± SEM (right) (unpaired *t*-test **P < 0.01). **b** Mock and p140 ACN cells. p130Cas phosphorylation was evaluated with antibodies to phosphorylated p130Cas at Tyr 410, p130Cas and GAPDH antibodies for loading control. Quantification on the right is the ratio between phosphorylated p130Cas and total p130Cas protein from three independent experiment, as mean ± SEM (right) (unpaired *t*-test ***P < 0.001). **c** Mock and p140 ACN cells. STAT3 phosphorylation was evaluated with antibodies to phosphorylated STAT3 at Tyr705 and STAT3 antibodies for loading control. Quantification on the right is the ratio between phosphorylated STAT3 and total STAT3 protein from three independent experiment, as mean ± SEM (right) (unpaired *t*-test ***P < 0.001). **d** Mock and p140 ACN cells. Jak2 activation was evaluated with antibodies to phosphorylated Jak2 at Tyr1007/1008) and Jak2 antibodies for loading control. Quantification on the right is the ratio between phosphorylated Jak2 and total Jak2 protein from three independent experiment, as mean ± SEM (right) (unpaired *t*-test ***P < 0.001). **e–g** SH-5Y-SY cells were transfected with a pool of p140Cap specific siRNA (si-p140) or the appropriate siRNA control (Ctr). At 72 h cells were extracted and tested for p140Cap WB, and GAPDH as loading contro (**e**), for Src activation at Tyr 416 phosphorylation (Y416) and Src protein level as loading control (**f**), and for phosphorylated STAT3 at Tyr 705 and STAT3 protein level as loading control (**g**). Quantification is shown on the left as the ratio between phosphorylated Src/STAT3 and total Src/STAT3 protein from three independent experiment, as mean ± SEM (right) (unpaired *t*-test *P < 0.05; **P < 0.01). **h** Bcl2 expression upon apoptotic stimulus was evaluated by WB of Mock and p140 cells, kept in suspension for 12 h (apoptotic stimulus) or in standard culture (basal conditions). Vinculin antibodies were used for loading controls. Quantification on the right is the ratio between Bcl2 and Vinculin protein from three independent experiment, as mean ± SEM (unpaired *t*-test ***P < 0.001). **i** Annexin V levels were assessed by a Flow cytometry assay to detect the percentage (%) of apoptotic cells in mock and p140 cells, kept in suspension for 24 h (apoptotic stimulus) or in standard culture (basal conditions) (unpaired *t*-test *P < 0.05). **j** p140 cells in apoptotic stimulus as in H were evaluated for STAT3 phosphorylation. Quantification on the right is the ratio between phosphorylated STAT3 (pSTAT3) and total STAT3 protein from three independent experiment, as mean ± SEM (unpaired *t*-test *P < 0.05)

Src kinase overexpression in NB patients has been associated with poor outcomes [30]. Conversely, its inhibition results in decreased proliferation and enhanced apoptosis in NB cells [31, 32]. The tyrosine phosphorylation of Src Tyr 416 (p-Src), a marker of active Src, as well as of p130Cas, a well-known Src substrate [33], were down-modulated in p140 cells, compared to mock cells (Fig. 3a, b). IL-6-dependent activation of STAT3 [34] has already been reported in NB, where STAT3 is critical in mediating increased survival and drug resistance [35–37]. In p140 cells, both STAT3 Tyr 705 (pSTAT3) and its upstream tyrosine kinase JAK2, were less phosphorylated (Fig. 3c, d).

p140Cap silencing with a specific pool of siRNA [17] resulted in increased phosphorylation of Src and STAT3 in SH-SY-5Y cells (Fig. 3e–g), further validating that p140Cap can control these pathways. Of note, p140Cap was not able to fully counteract the high phosphorylation of a constitutively active STAT3 (STAT3C) [23], or Src (SrcY527F) (Supplementary Fig. S2a, S3a). Overall, p140 ability to influence the Src/p130Cas and the JAK2/STAT3 pathways could be causal for the impairment of NB progression.

Based on the pro-survival role of STAT3 in NB [35, 38], we performed apoptosis assays upon matrix cell detachment, or anoikis, with cells kept in suspension for 12 h. In p140 cells, the up-regulation of the antiapoptotic marker Bcl2 [39] was limited to a 2,9 fold, while in mock cells it reached an 8 fold increase (Fig. 3h). Indeed, both mock and p140 transduced cells showed an increase in Annexin V staining upon anoikis, with a significant increased percentage of apoptosis marker in p140 cells (Fig. 3i), indicating that p140Cap limits the ability of NB cells to induce survival pathways. p140 cells showed also significant decrease in the level of pSTAT3 in the anoikis conditions over mock cells (Fig. 3j). Only the presence of the constitutive active STAT3C mutant rendered p140 cells less sensitive to anoikis-dependent death (Supplementary Fig. S2c). Overall, these results suggest that the presence of p140 is pro apoptotic by impairing signaling through the JAK2/STAT3-Bcl2 survival pathway.

### p140Cap dampens in vivo tumor growth and spontaneous metastasis formation

**Fig. 4:**
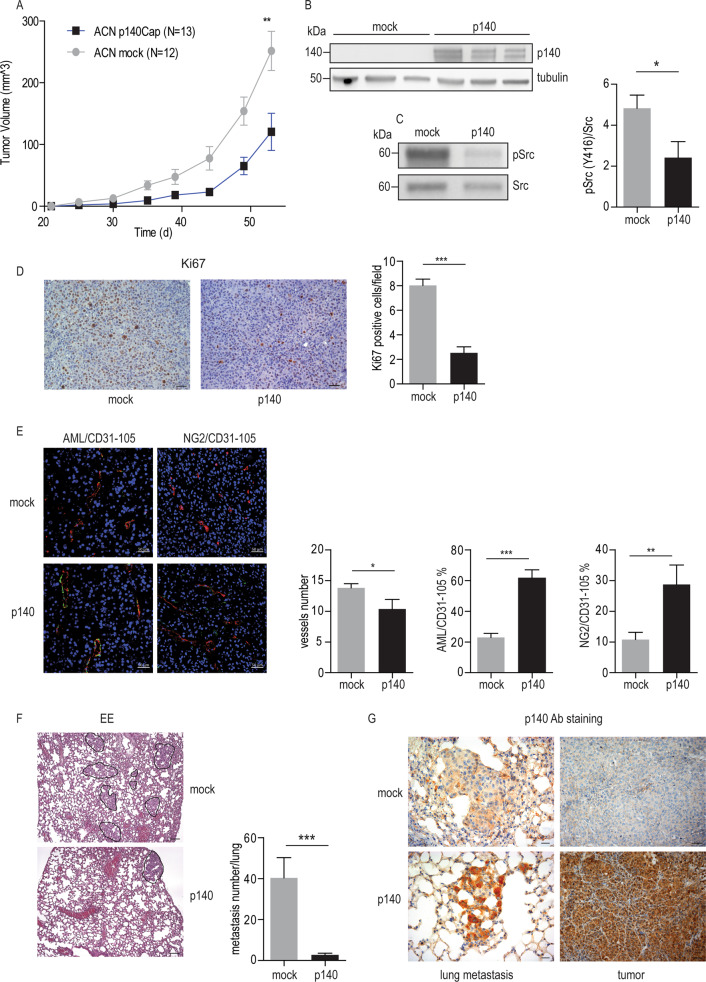
p140Cap limits in vivo tumor growth and spontaneous metastasis formation. Mock and p140 cells (2 × 10^5^ cells in 0.2 ml of PBS) were subcutaneously injected into the dorsal region of male NSG mice. **a** Average tumor volume. The size of the tumors was evaluated twice a week using digital calipers in blind experiments and significance was quantified by unpaired t-test (**P < 0.01). **b** WB analysis of p140Cap expression. Extracts from explanted tumors were analysed on a 4–15% precasted SDS-PAGE. Antibodies to p140Cap and tubulin (as loading control) were used. **c** Src kinase activation state in tumor extracts. Representative WB analysis of Tyr 416 phosphorylation (Y416) and Src protein level is shown. Quantification on the right is the ratio between phosphorylated Src and total Src protein in 5 tumors per group, as mean ± SEM (right) (unpaired *t*-test **P < 0.01). **d** Ki67 proliferation marker on tumors sections. Representative Ki67 staining of tumors sections in the left panel and percentage of positive cells (number of Ki67 positive cells/total cells x 100) for x200 field (unpaired *t*-test ***P < 0.001). Scale bar 50 µm. **e** Pericyte coverage evaluation. Immunofluorescence images by confocal microscopy of tumors stained with CD31-105 (red) for endothelia and AML (green, left column) for mature pericytes or NG2 (green, right column) for young pericytes. The number of vessels in x200 fields are quantified in the left hystogram. Quantifications of the extent of pericyte coverage on endothelia are shown on middle and right hystograms (unpaired *t*-test; *P < 0.05; **P < 0.01; ***P < 0.001). Scale bar 100 µm. **f** Spontaneous lung metastasis formation. Hematoxylin and eosin sections of lung tissue derived from NSG mice bearing mock or p140-tumors. Quantification of spontaneous lung micro-metastases is shown in the right histogram (unpaired *t*-test ***P < 0.001). Scale bar 100 µm. **g** p140Cap expression in lung metastasis and tumors. Representative images of lung metastasis (left) and primary tumors (right) from NSG mice bearing mock or p140 primary tumors stained with p140Cap antibodies. Scale bar 20 µm (left) and 50 µm (right)

Based on the association between p140Cap status and increased overall free-survival in NB patients, we addressed the putative tumor-suppressing role of p140Cap in NB in the in vivo setting, analyzing both tumor growth and metastatic events. When mock and p140 transduced cells were injected subcutaneously in the dorsal region of immunodeficient NOD Scid Gamma (NSG) mice, tumors derived from p140 cells showed a delay in palpable tumor formation (from 30 to 35 days), accompanied by a significant reduction in growth, compared to tumors from mock cells (Fig. 4a). p140Cap was still expressed in explanted tumors derived from p140 cells (Fig. 4b). Consistent with in vitro observations, p140 tumors showed lower levels of active Src over mock tumors (Fig. 4c).

Immunohistochemistry analysis on tumor sections confirmed that the proliferation marker Ki67 was widely more expressed by mock tumors cells, compared to p140 tumors (Fig. 4d). The tumor vasculature was stained with both CD31-105 endothelial cell markers and AML or NG2, markers of mature or young pericytes, respectively [40]. While the number of pixel stained in red with the CD31-105 endothelial cell marker was similar between mock and p140 tumors, vessel count showed a slight but significant decrease in p140 tumors (Fig. 4e). Consistent with the idea that p140 restrains the angiogenic activity of tumor cells leading to the formation of larger and more stable vessels, both AML and NG2 staining showed significantly higher pericyte coverage of endothelia in p140 versus mock tumors.

The association between p140Cap and increased survival in NB patients, and the different vessel morphology, lead us to compare the spontaneous metastatic process from ‘same size' mock and p140 primary tumors. Micro-metastasis were observed only in lungs, and not in liver, kidney, lung and femurs. However, p140 tumors gave rise to a significantly reduced number of lung metastases over mock tumors (Fig. 4f). p140Cap staining of lung metastases in p140 tumors showed that metastatic cells were still positive for p140Cap (Fig. 4g).

Overall, p140Cap impairs NB tumor growth and spontaneous metastasis in vivo, with a significant decrease in proliferation markers and an increase in tumor vessel pericyte coverage.

### p140Cap increases NB cell sensitivity to chemotherapeutic treatment

**Fig. 5:**
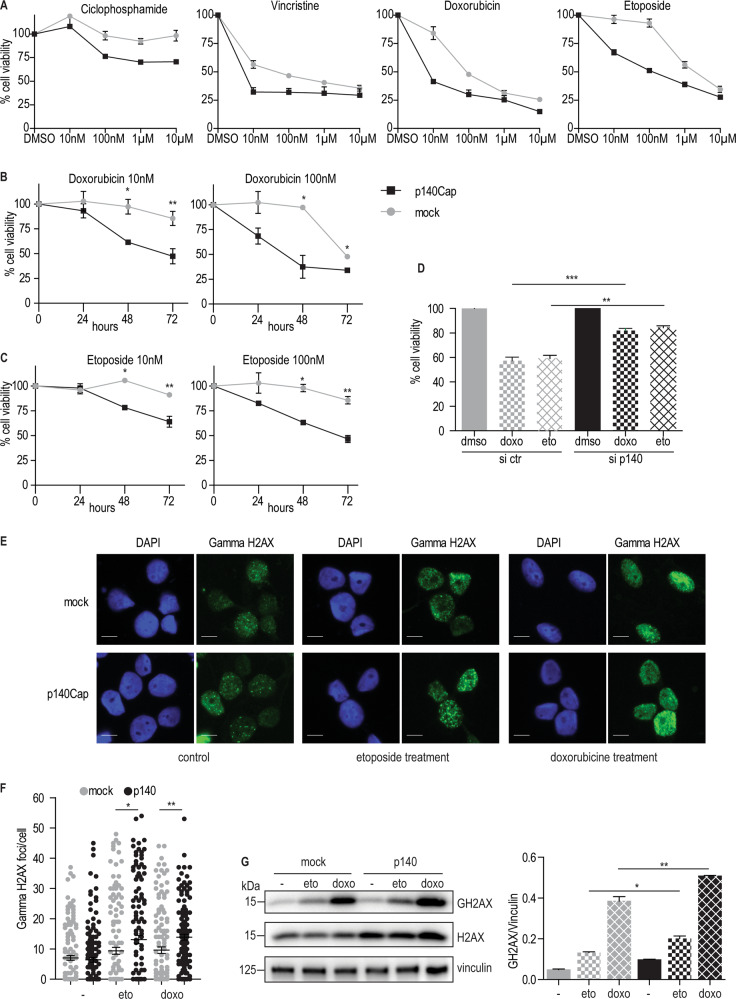
p140Cap regulates cell viability to chemotherapeutic drugs. **a**) Dose dependence viability. Mock and p140 cells were plated in 24-well plates and treated with four different doses of the indicated drugs (10 nM, 100 nM, 1 µM, 10 µM). Cell viability was quantified at 72 h of treatment with cyclophosphamide, vincristine, doxorubicin, or etoposide at the indicated concentrations or with DMSO as control, after staining with Crystal Violet solution. Absorbance was read at 570 nm. Results show 3 independent treatment as mean + SEM (*P < 0.05; **P < 0.01). **b**, **c** Time dependent viability. Mock and p140 cells were processed as in (**a**) in response to two low doses (10 nM and 100 nM) of doxorubicin (**b**) and etoposide (**c**) for 24, 48 or 72 h. Results show three independent treatment as mean + SEM (*P < 0.05; **P < 0.01). **d** Cell viability in SH-5Y-SY cells silenced for p140Cap. Cells were transfected with appropriate siRNA and after 24 h treated with 1 µM etoposide or doxorubicin. Cell viability was quantified at 48 h. Results show three independent experiments as mean + SEM (**P < 0.01; ***P < 0.001). **e** Visualization of nuclear foci for gamma H2AX histone as a marker of DNA damage. Mock and p140 cells were plated on glass slides for 48 h, starved overnight and treated for 6 h with 1 µM etoposide and doxorubicin. Cells were processed as in Material and Methods. Representative images are shown. Green: gamma H2AX foci; Blue: DAPI for nuclear staining. Scale bar: 10 µm. **f** Quantification of gamma H2AX foci/cell. Mock: red; p140: green. 50 nuclei were evaluated for each experiment. Experiments were performed in triplicates (unpaired t-test and Welch’s unequal variance *t*-test, *P < 0.05; **P < 0.01). **g** Gamma H2AX levels upon chemotherapy treatment. Mock and p140 cells were plated in 6-well plates and acutely treated with 1 µM etoposide or doxorubicin for 6 h. Extracts were run on 4–15% pre-casted SDS-PAGE and analyzed with antibodies to gamma-H2AX, H2AX and vinculin for loading controls. Quantification on the right is the ratio between gamma-H2AX and vinculin, from three independent experiment, as mean + SEM (right) (unpaired *t*-test *P < 0.05; **P < 0.01)

To assess whether the increased survival of p140-expressing NB patients might correlate with increased cell sensitivity to chemotherapy, five chemotherapeutic drugs commonly used in NB patients (ciclophosphamide, carboplatin, doxorubicin, etoposide, and vincristine) [5, 41] were tested in dose viability assays. p140 cells showed significantly increased sensitivity to low doses (10, 100 nM) of cyclophosphamide, vincristine, doxorubicin and etoposide (Fig. 5a), but not carboplatin (data not shown). Time course analysis were performed with doxorubicin and etoposide, two drugs used in first line treatment of NB, at low doses. For both drugs, p140 cells showed increased sensitivity already at 48 h over mock cells (Fig. 5b, c). Consistently, in SH-SY-5Y cells, p140Cap silencing resulted in increased viability to both doxorubicin and etoposide (Fig. 5d).

Both etoposide and doxorubicin prevent ligation of the DNA strands, stopping the process of replication. To assess whether the increased sensitivity of p140 cells to these drugs was associated with increased DNA lesions, the number of foci/cells of phosphorylated histone H2AX (gamma H2AX), an established marker of DNA damage [42], were quantified after an acute 6 h treatment with 1 µM etoposide and doxorubicin. This marker was significantly increased in p140 cells over mock cells (Fig. 5e, f), as shown by immunofluorescence and WB analysis of phosphorylated gamma H2AX (Fig. 5g).

Overall, p140 cells display a significant decrease in cell viability, with an increased sensitivity to drug-dependent DNA damage.

### p140Cap sensitizes cells to combined treatment with chemotherapy and Src kinase inhibitors

**Fig. 6:**
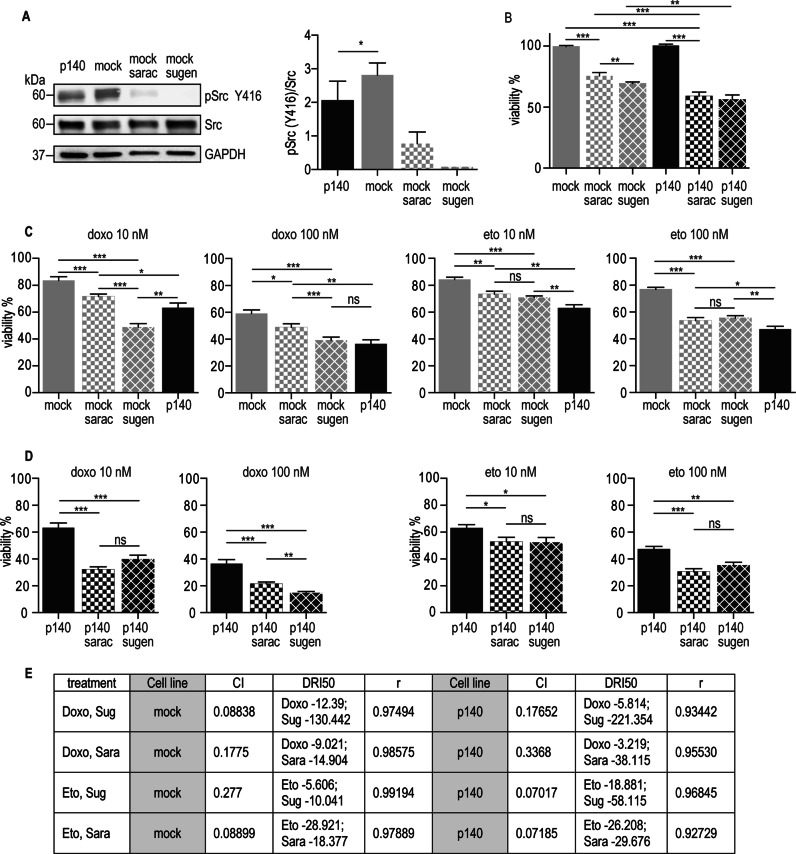
p140Cap sensitizes cells to combined treatments with chemotherapy drugs and Src kinase inhibitors. **a** Effect of Src inhibitors on Src phosphorylation in Mock cells. Mock and p140 ACN cells were plated in 24-well plates and treated with saracatinib (100 nM) and sugen (1 µM) or DMSO used as control. Src activation was evaluated by WB analysis of Tyr 416 phosphorylation (Y416). WB for Src and GAPDH proteins was used as loading control. Quantification on the right is the ratio between phosphorylated Src and total Src protein from three independent experiment, as mean + SEM (right) (unpaired *t*-test *P < 0.05). **b** Effect of Src inhibitors on cell viability. Mock and p140 ACN cells were plated in 24-well plates and treated with saracatinib (100 nM) and sugen (1 µM) or DMSO used as control. Cell viability was quantified at 72 h by staining with Crystal Violet solution. Absorbance was read at 570 nm. Results is presented for three independent experiments as mean ± SEM (unpaired *t*-test **P < 0.01; ***P < 0.001). **c** Combined treatment with doxorubicin/etoposide and Src inhibitors in mock cells. Cells were plated in 24-well plates and treated as indicated. Cell viability was quantified as in (**b**). p140 cells were used as control. 100% viability (not shown in the Figure) refers to the viability of untreated mock cells. Results is presented for three independent treatment as mean ± SEM (unpaired *t*-test *P < 0.05; **P < 0.01; ***P < 0.001). **d** Combined treatment with doxorubicin/etoposide and Src inhibitors in p140 cells. p140 cells were plated in 24-well plates and treated as indicated. Cell viability was quantified as in A). Viability of untreated p140 cells was expressed as percentage of viable cells towards untreated mock ACN cells. 100% viability (not shown in the Figure) refers to the viability of untreated p140 cells. Results is presented for three independent treatment as mean ± SEM (unpaired *t*-test *P < 0.05; **P < 0.01; ***P < 0.001). **e** Dose-response assays for synergistic analysis. The CalcuSyn software (www.biosoft.com/w/calcusyn.htm) was used to calculate the CI (combination index to reduce viable cells to 50%) and the DRI50 (the dose-reduction necessary to decrease viable cells to 50%); r: linear regression coefficient

Since active Src is significantly down-regulated in p140 cells and tumors (see Figs. 3a, 4c), we assessed whether Src activity may be involved in NB cell viability. In mock cells, Src activity was highly sensitive to two well-known Src inhibitor, saracatinib (which also inhibit the Abl kinase [43]) at 100 nM, and sugen (used in preclinical NB models [44]) at 1 μM (Fig. 6a). At 72 h, in mock cells both inhibitors decreased cell viability of 20–25%. Interestingly, the same treatment in p140 cells leads to a stronger reduction in viability of 40% (Fig. 6b). Moreover, viability to Src inhibitors was increased in cells partially silenced for p140Cap (70% decreased expression) compared to p140 cells. However, the partially silenced cells were still more sensitive than mock cells (Supplementary Fig. S4a, b), suggesting that p140Cap level directly correlates with increased sensitivity to Src inhibitors.

A combined treatment with genotoxic drugs and Src inhibitors (10 nM and 100 nM etoposide or doxorubicin in combination with sugen and saracatinib) decreased mock cell viability to the levels observed in p140 cells treated with doxorubicin or etoposide alone (around 50% viability, see Fig. 5b) (Fig. 6c), suggesting that Src inhibition may contribute to enhance chemotherapy cytotoxicity in cells that do not express p140Cap. Moreover, the combined treatment on p140 cells further significantly reduced cell viability, especially in doxorubicin-treated cells (Fig. 6d).

Overall, sensitivity of NB cells to doxorubicin and etoposide is further enhanced by the combined treatment with Src inhibitors. To clarify whether this enhancement was due to an additive or a synergistic effect, we performed dose-response experiments, incubating mock and p140 cells with increasing concentrations of doxorubicin and etoposide (from 1 nM to 1 mM), alone or in combination with the same concentration range of Src inhibitors. The combined treatment was synergistic both in mock and p140 cells, because the Combination Index (CI) values computed for the different combinations of drugs were <1 in all the experimental settings (Fig. 6e; Supplementary Fig. S2) [24]. Dose reduction index 50 (DRI50) quantifies the extent of dose reduction obtained for the 50% viability inhibitory effect in combination setting in comparison to each drug alone. The p140 cells still showed an increased sensitivity to the Src inhibitors in the combined treatment, with a shift of the sensitivity to lower dose than in mock cells (Supplementary Fig. S2, right panels). Therefore, chemotherapy and Src inhibitors combination synergistically decreases NB cell viability and this effect can be further increased by p140 expression.

## Discussion

The data reported here are consistent with an onco-suppressive function of *SRCIN1*/p140Cap in NB tumors. *SRCIN1* gene expression strongly correlates with good outcomes in NB, due to the ability of the p140Cap protein to negatively regulate molecular pathways exploited for tumor progression. High levels of *SRCIN1* mRNA are clinically relevant in NB patients, positively correlating with good prognosis and high survival rate, both OS and EFS, meaning that *SRCIN1* expression correlates with decreased metastatic recurrences in NB patients. *SRCIN1* expression is a risk factor independent from the other known risk factors, such as *MYCN* oncogene amplification, INSS stage and age at diagnosis, recognized as the strongest indicators of aggressive tumor behavior in NB patients. Thus, *SRCIN1* could provide a useful, additional marker for better stratifying NB patient cohorts. Moreover, p140Cap is expressed in the human neonatal adrenal medulla, the primary site for NB onset in over 30% of patients [2].

The *SRCIN1* gene is located on chromosome 17q12, a genomic region frequently involved in genetic abnormalities in NB. In 17 out of 225 NB patients, the *SRCIN1* gene status is altered, suggesting putative modification of *SRCIN1* gene expression in NB patients. The presence of translocation breakpoint involving 17q interrupting the *SRCIN1* gene, hemizygously deletion, or cn-LOH have been detected in primary tumors of all stages with 17q gain, which associates with NB poor prognosis. *SRCIN1* gene status may affect p140Cap expression, as shown in SK-N-SH cells, where cn-LOH (8.72 Mb) that included *SRCIN1* gene, correlates with weak protein expression, suggesting an effective partial knockout of gene expression.

Overall, p140Cap acts as a tumor suppressor gene in NB tumors, decreasing tumor growth and reducing probability of developing distant metastasis. The increased apoptosis observed upon anoikis and the reduced Ki67 levels suggest a different sensitivity of p140 cells to the in vivo growth. Moreover, p140 tumor vessels may exhibit reduced permeability, likely thanks to the increase of the pericyte marker NG2 on tumor vessels [40]. These data are consistent with those obtained in HER2-related breast cancer preclinical models [17], where p140Cap limits the aggressiveness of HER2-related tumors, increasing both sensitivity to apoptosis and tumor differentiation.

We have previously reported that p140Cap attenuates Src kinase activity in breast cancer cells upon integrin-mediated adhesion or growth factor treatment stimulation [18, 45], while absence of p140Cap leads to enhanced Src activity in vivo in synaptosomes from p140Cap KO mice [20]. In NB tumors p140Cap impairs both Src and STAT3/Jak2 pathways, key for NB growth and drug resistance [30, 31, 35–38]. Also in the presence of Src or STAT3 ‘constitutive active' forms, the p140 cells are still less aggressive than mock cells in anchorage-independent growth, suggesting a key role for p140Cap in regulating these signaling mechanisms. The ability of p140Cap to down-regulate Src activity is thus a common feature for at least two cancer subtypes and may be a key event in dampening their migratory and invasive phenotype.

Despite progress made in developing new therapies, the 5-year overall survival rate of high-risk NB patients remains less than 40% [46]. The therapeutic efficacy of commonly NB treatments (e.g., Vinca alkaloids, platinum drugs, and anthracyclines) is often hampered by the poor bioavailability of the drug at the site of action [47], which decreases sensitivity. Moreover, survivors of NB frequently encounter lifelong health issues from the therapy they received as a child. p140 cells show significantly increased sensitivity to low doses (10 nM concentration) of doxorubicin and etoposide, two drugs used in first line NB treatment. Only expression of a constitutively active Src kinase may partially overcome p140Cap sensitivity to these drugs (Supplementary Fig. S3b). Overall, p140Cap may have prognostic value, in terms of a potential reduction of the cytotoxic effects of chemotherapy, one of the main issue for pediatric tumor treatment [48]. Moreover, the combination of Src inhibitors with doxorubicin or etoposide, sensitize mock cells, reducing cell viability to that of p140 cells treated with chemotherapy alone. Src inhibitors and chemotherapy exert together a synergistic effect, more pronounced in p140 cells. The DRI50 levels show that the p140 cells are more susceptible to Src inhibitors compared to mock cells, opening the way to possible new combinations of treatments in the p140-expressing NB patients.

p140Cap cells show increased sensitivity to drug-dependent DNA damage. Recently, the Src family kinases (SFK) have been shown to promote recovery from G2 DNA damage checkpoint arrest by inhibiting ATR-Chk1 signaling in doxorubicin-treated cells. Inhibition of SFK activity leads to persistent checkpoint activation and prolonged cell cycle arrest [49, 50], providing a possible explanation on the observed greater efficacy of combined Src inhibitors and DNA damaging agents over the treatment alone. As p140Cap can interact with multiple effectors in the synapse [51], in addition to Src activity downregulation, p140Cap ability to associate proteins belonging to multiple pathways can contribute to the increased sensitivity of p140 cells to combined treatments.

Besides genomic translocations, deletions or cn-LOH, additional mechanisms can account for alteration of *SRCIN1* expression in NB patients. Data on the regulation of expression of p140Cap gene are currently limited to miRNAs [52, 53] able to impair epithelial cancer cell features via the inhibition of *SRCIN1* expression. Our recent work show that in HER2-related breast cancer, amplification of the *ERBB2* locus may lead to *SRCIN1* amplification [17], thus contributing to the biological heterogeneity of this breast cancer subgroup [54].

Altogether, these data highlight the potential clinical impact of *SRCIN1*/p140Cap expression and of p140Cap-regulated pathways in NB tumors as a new player extremely relevant for patient outcome.

## Supplementary information

Supplemental material
