# Supplementary material for: The SRCIN1/p140Cap adaptor protein negatively regulates the aggressiveness of neuroblastoma
Source: Cell Death Differ. 2019 Jul 8;27(2):790–807. doi: 10.1038/s41418-019-0386-6 (PMC7205889; doi:10.1038/s41418-019-0386-6)

**Supplementary Table 1. *SRCIN1*, *ERBB2* status on NB cell lines**

| NB CELL LINES | *SRCIN1* | CHROMOSOMAL COORDINATES OF *SRCIN1* LOSS/GAIN | *ERBB2* | CHROMOSOMAL COORDINATES OF *ERBB2* LOSS/GAIN |
| --- | --- | --- | --- | --- |
| ACN | loss | Chr17: 27758517-38185359  Cytoband: 17q11.2-q21.1  Size: 10.42 Mb | loss | Chr17: 27758517-38185359  Cytoband: 17q11.2-q21.1  Size: 10.42 Mb |
| SH-SY5Y | single copy |  | single copy |  |
| SK-N-SH | copy neutral LOH | Chr17: 32828526-41555474  Cytoband: 17q12-q21.31  Size: 8.72 Mb | copy neutral LOH | Chr17: 32828526-41555474  Cytoband: 17q12-q21.31  Size: 8.72 Mb |
| SK-N-BE(2)c | single copy |  | single copy |  |
| LAN-1 | gain | Chr17: 31257852 -81029941  Cytoband: 17q11.2-q25.3  Size: 49.77 Mb | gain | Chr17: 31257852 -81029941  Cytoband: 17q11.2-q25.3  Size: 49.77 Mb |
| LAN-5 | gain | Chr17: 35862059 -81029941  Cytoband: 17q12-q25.3  Size: 44.16 Mb | gain | Chr17: 35862059 -81029941  Cytoband: 17q12-q25.3  Size: 44.16 Mb |
| IMR32 | single copy |  | single copy |  |
| HTLA-230 | single copy |  | single copy |  |

**Supplementary Figure S1: Status of *SRCIN1* and *ERBB2* genes in ACN and SK-N-SH cell lines.**


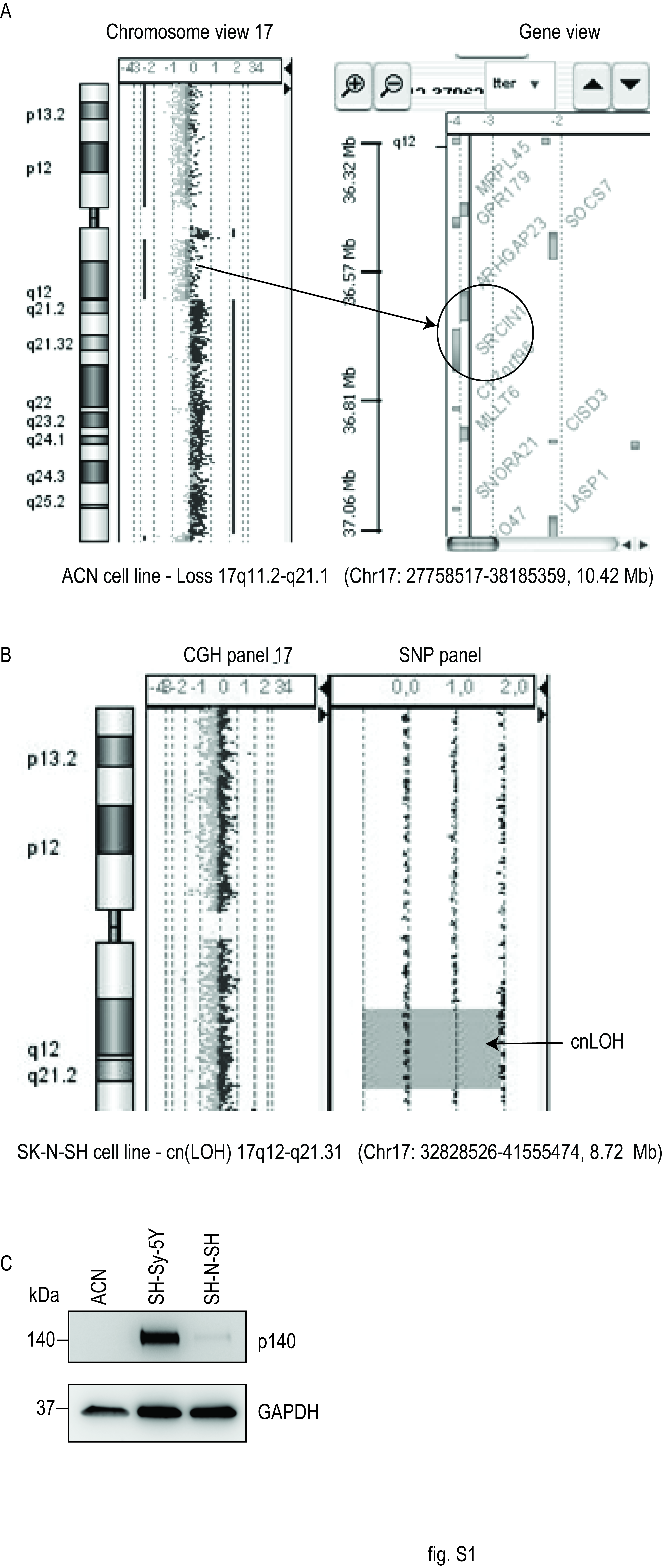


**Supplementary Figure S2. Expression of a constitutive active STAT3 partially interferes with p140Cap effects on growth and anoikis.**

**
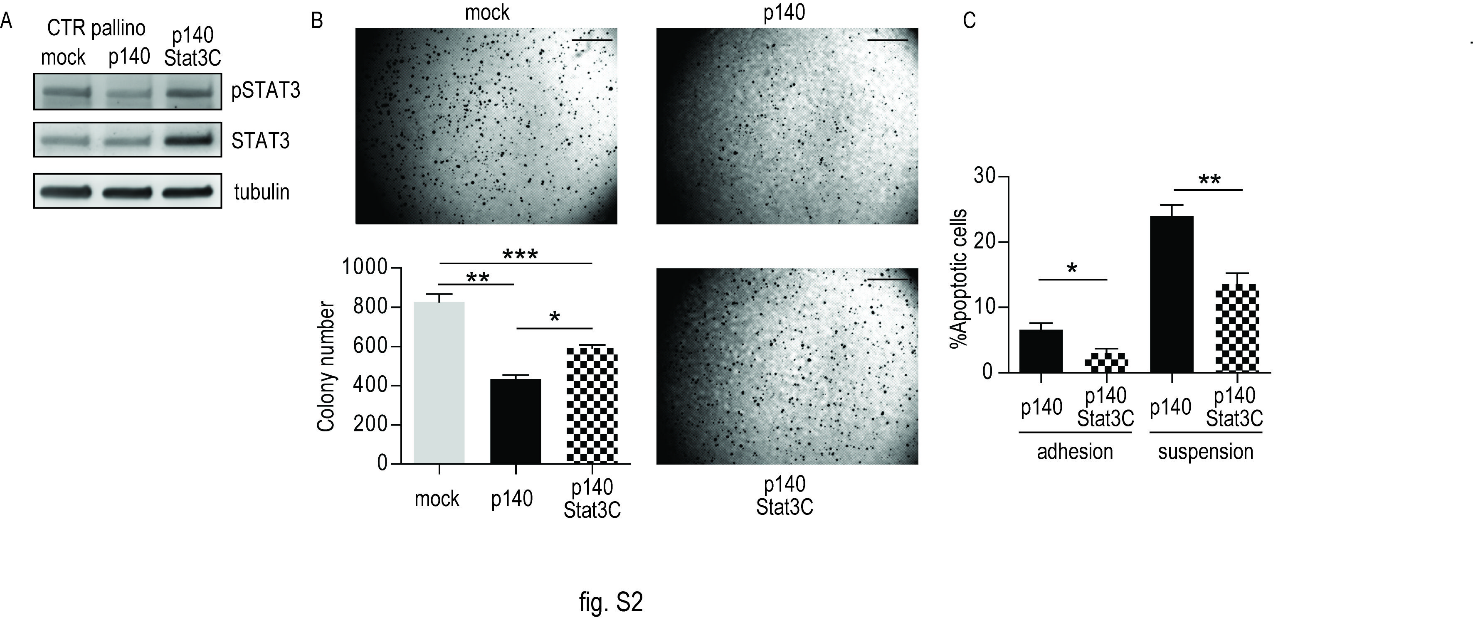
**

Cells expressing p140 were infected with a retrovirus construct (Pallino = retrovirus construct LZRSpBMN-Z-GFP) expressing constitutive active STAT3, as described in [23]. The bulk population was screened for expression by WB (panel A) with antibodies to STAT3 and to pSTAT3-Y705. These cells were subjected to anchorage-independent growth assay (panel B), and anoikis assay (panel C) (unpaired t-test *P<0.05, **P<0.01, ***P<0,001).

**Supplementary Figure S3.** **Expression of a constitutive active Src kinase decreases p140 cell sensitivity to doxorubicin and etoposide.**

**
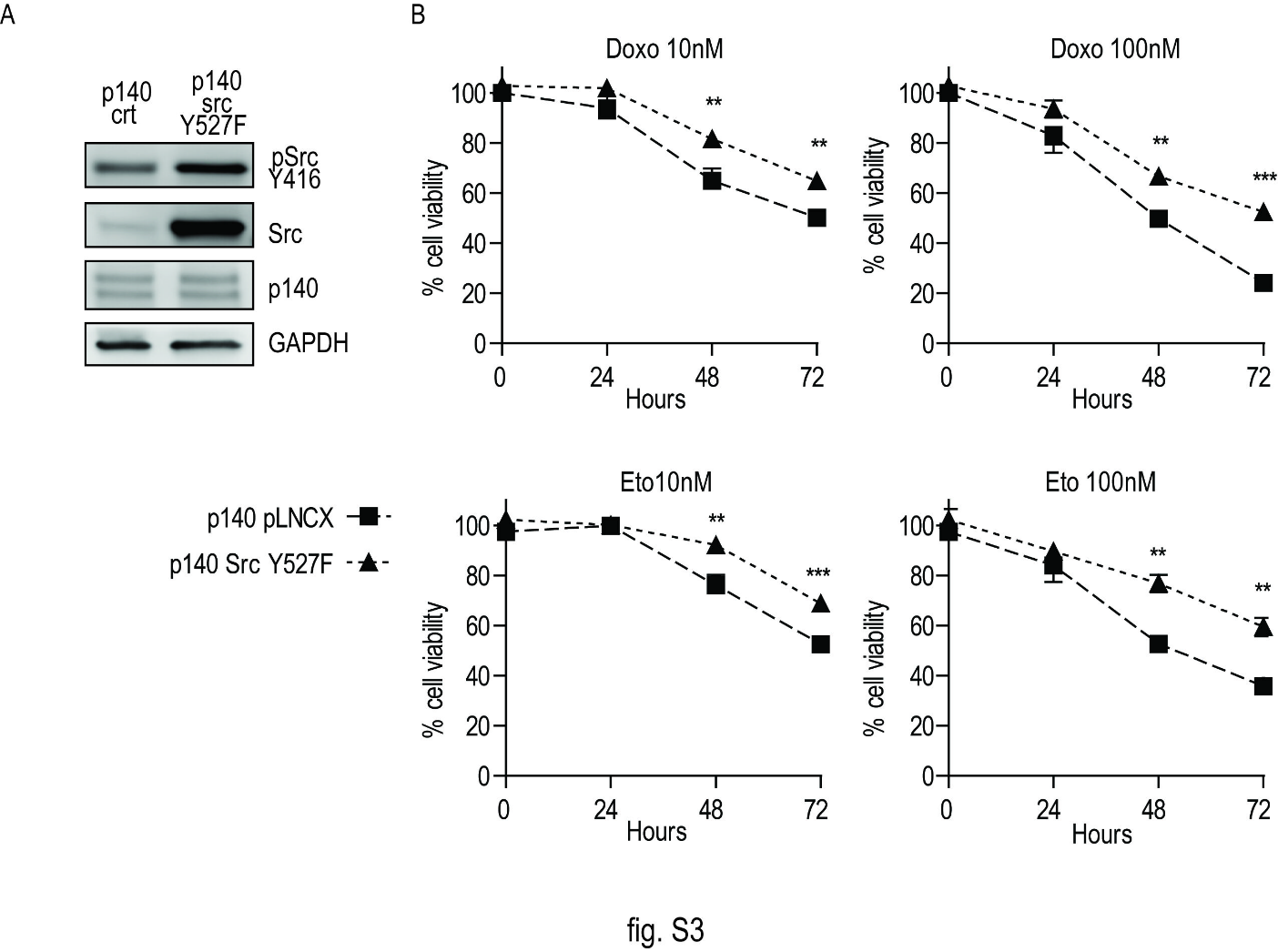
**

Cells expressing p140Cap were transfected with a construct expressing constitutive active Src (Src Y527F, from Addgene , Plasmid #13660). A pool of selected clones was screened for expression by western blot (panel A) with antibodies to pan-Src and to pSrc-Y416. The same cells were subjected to viability assays upon treatment with 10 and 100 nM doxorubicin (Doxo) or etoposide (Eto) (panel B). Results show 3 independent experiments as mean + SEM (unpaired t-test **P<0.01, ***P<0,001).

**Supplementary Figure S4. Correlation between p140Cap expression and sensitivity to Src inhibitors.**

**
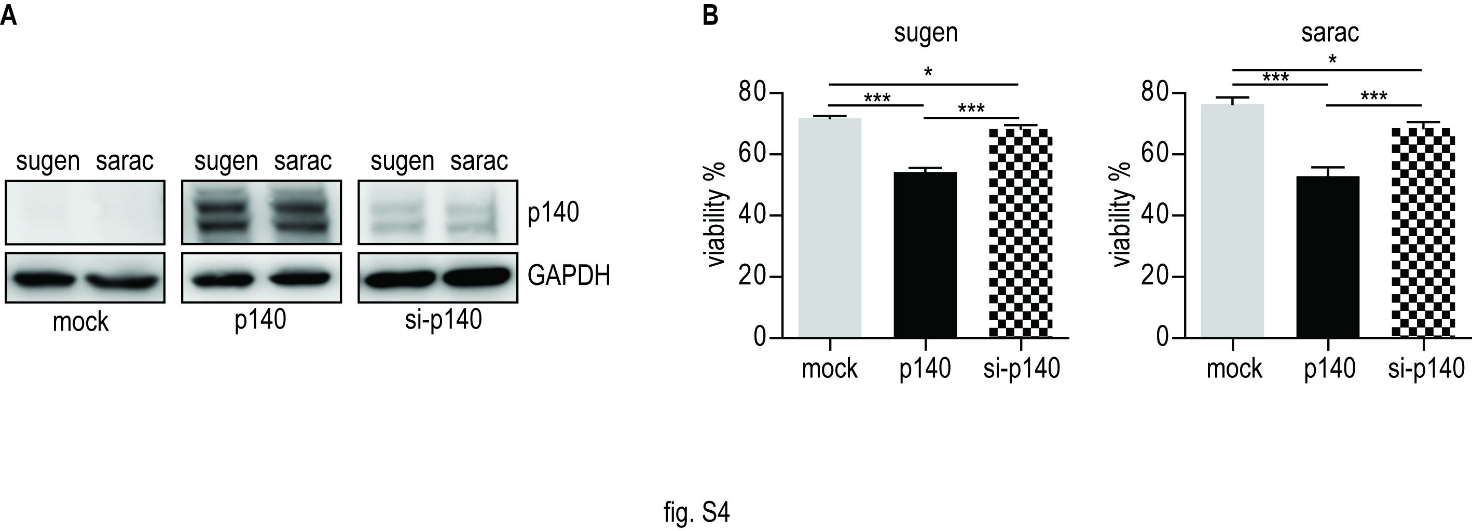
**

p140Cap was partially silenced in p140 cells, reaching a 70% decrease of protein expression (panel A). Mock, p140 and p140-silenced cells were treated with 1μM sugen or 100 nM saracatinib in viability assays for 72 hours (panel B). Results show 3 independent experiments as mean + SEM (unpaired t-test *P<0.05, ***P<0,001).

**Supplementary Figure S5. Dose-response curve of combined treatments with doxorubicin/etoposide and Src inhibitors.**


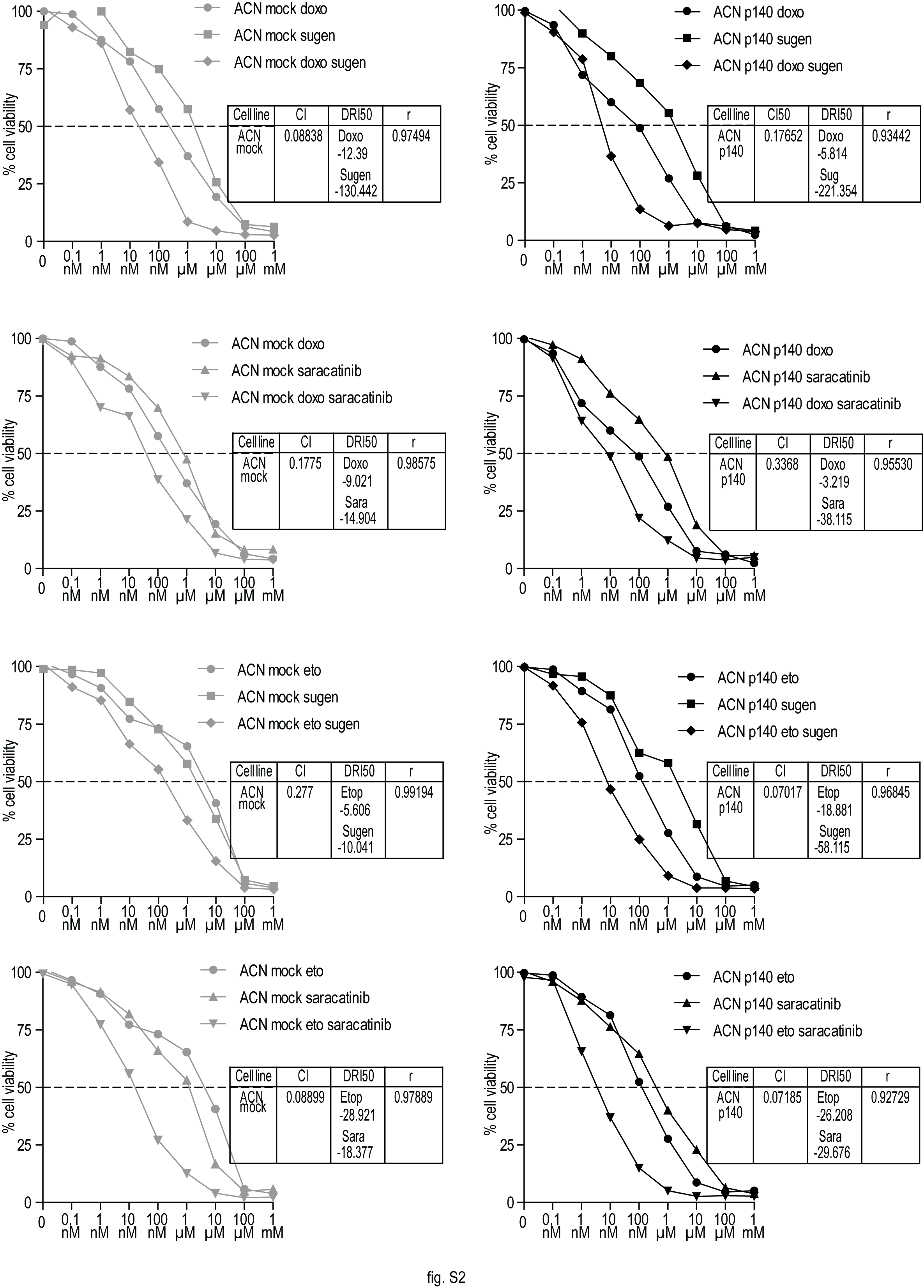

Supplement: Supplementary file 1 — Supplemental material [file 41418_2019_386_MOESM1_ESM.docx]
